# 

**Published:** 1897-09-25

**Authors:** 


					THE MUtSflTAL.
ABSOLUTELY PURE, therefore BEST. Sept. 25th, 1897. ^
CADBURY'S l?t CAI??co???
GOCOA Jnl Ifrll /M ' j j -p n y
" The standard of highest purity at pwiBiit t NO ALKALIES UsErD^ ^
attalnable."?Lancet. I G'JRG t ON G L N L R A L S
mwicH uoshljaj.
soon Wis TERM InFIRMAMT
No. 574. Vol. xxii. [J3ES5S2] Saturday,
Sept. 25th, 1897.
[SnhBoriptionTerniB,] pRICE TWOPENCE.

				

